# Localization performance of cochlear implant users with a real-time bilaterally-synchronized sound coding strategy that provides explicit interaural timing cues with mixed rates of stimulation

**DOI:** 10.3389/fnins.2025.1682452

**Published:** 2025-10-28

**Authors:** Agudemu Borjigin, Stephen R. Dennison, Alan Kan, Ruth Y. Litovsky

**Affiliations:** ^1^Binaural Hearing and Speech Lab, Waisman Center, University of Wisconsin-Madison, Madison, WI, United States; ^2^Department of Communication Sciences and Disorders, University of Utah, Salt Lake City, UT, United States; ^3^North American Research Laboratory, MED-EL US, Durham, NC, United States; ^4^School of Engineering, Macquarie University, Sydney, NSW, Australia

**Keywords:** bilateral cochlear implant, interaural time difference, localization, synchronization, mixed rates

## Abstract

**Introduction:**

Bilateral cochlear implants (BiCIs) do not restore sound localization abilities to the full extent exhibited by typical hearing (TH) listeners, partly due to poor encoding of interaural time differences (ITDs). ITD cues have been provided and investigated using synchronized research processors that ensure the precise delivery of ITD cues. These studies have been conducted in a direct stimulation setting, which bypasses the processor microphones and, in most cases, removes interaural level difference cues (ILDs). To our knowledge, this is the first study that evaluated the efficacy of synchronized stimulation in restoring sensitivity to ITDs in a free field localization experiment. This was made possible by the CCi-MOBILE, a portable and real-time processing research platform that allows for synchronizing microphone inputs.

**Methods:**

Fourteen BiCI listeners were tested with experimental real-time coding strategies in comparison to unsynchronized clinical processors. We calculated the binaural cues from the acoustic stimuli at the level of microphone input.

**Results:**

The recordings show that the experimental coding strategies in this study deliver ITDs with greater precision than the clinical strategy. However, psychophysical testing did not show the benefit of an ITD-encoding strategy in improving localization in a free field. The ITD encoding strategies preserved ITDs, which better differentiated unique loudspeaker locations than interaural level differences (ILDs), suggesting that listeners could achieve improved performance if they accessed these cues. As expected, ILDs were similar across all strategies, including the ITD encoding strategies. The lack of improvement in localization performance is likely because ILDs remained to be the dominant cue in acute localization testing, even when ITD cues were available.

**Discussion:**

Providing BiCI listeners with adequate experiences with ITD cues may be necessary to shift their reliance from ILD dominance to a combined reliance on ILD and ITD cues in free-field conditions. The CCi-MOBILE could enable take-home practice with novel stimulation strategies for extended experiences and long-term evaluation in real-world listening environments.

## 1 Introduction

Sound localization is a critical hearing ability for everyday listening, especially when there are multiple sound sources. The localization of sound in the horizontal plane relies on the detection of interaural time differences (ITDs) and interaural level differences (ILDs). ITDs and ILDs arise from physical differences in sound intensity and arrival time between listener’s ears, respectively (Rayleigh, 1909). For people with bilateral moderate to profound deafness, bilateral cochlear implants (BiCIs) can grant access to ITDs and ILDs by restoring the perception of sound to both ears (Brown and Balkany, 2007; Kan and Litovsky, 2015; van Hoesel and Tyler, 2003). However, clinically available BiCIs do not restore sound localization ability to the same level as typical hearing listeners (TH) (Dorman et al., 2016), and the outcome varies widely from patient to patient (Anderson et al., 2022). If a BiCI listener shows sound localization capability, they have been shown to rely primarily on ILDs (Aronoff et al., 2010; Dorman et al., 2014; Grantham et al., 2007; Kelvasa and Dietz, 2015) but not ITD cues (Kan and Litovsky, 2015; van Hoesel and Tyler, 2003).

BiCI listeners are likely unable to fully access ITD cues for several reasons, some of which we address here. In this study, we focus on the following two technological limitations of commercially available devices. First, clinical processors are not synchronized across the ears, which can introduce uncontrolled timing variations of up to hundreds of microseconds between pulse timing across the two ears (Dennison et al., 2022; Gray et al., 2021). This timing delay can be problematic considering that the maximum ecologically relevant ITD is around 700 μs (Moller et al., 1995). Second, most clinically available CI sound coding strategies extract only the envelopes of sounds and use them to modulate electrical pulse trains with high stimulation rates. The stimulation rate of pulse trains is typically high at around 1,000 pulses per second (pps) to accurately represent envelopes (Loizou et al., 2000). Although high-rate stimulation is important for speech intelligibility, the sensitivity of BiCI listeners to ITD has been shown to be better at low stimulation rates (Anderson et al., 2019; Carlyon et al., 2025; Kan and Litovsky, 2015; Laback et al., 2007; van Hoesel et al., 2009; van Hoesel and Tyler, 2003). Coding strategies such as MED-EL (Innsbruck, Austria) fine structure processing algorithms can provide lower stimulation rates by matching pulse timing to zero crossings in the most apical or low-frequency channels (Hochmair et al., 2015). However, with these strategies, most channels will not receive stimulation at a sufficiently low rate for optimal ITD sensitivity, and lack of synchronization can inhibit real-world benefits (Dennison et al., 2022).

Synchronized research platforms allow researchers to potentially overcome these two challenges by enabling the development and investigation of sound coding strategies with bilateral synchronization, ensuring the precise delivery of ITD cues at custom stimulation rates (Litovsky et al., 2017). Cochlear implant manufacturers can provide these research tools to the broader community, and indeed the Cochlear Nucleus Implant Communicator, Advanced Bionics BEDCS2, and University of Innsbruck RIB2 tools for MED-EL implants have facilitated many studies on ITD sensitivity (e.g., Laback et al., 2015). However, such research platforms lack integration with behind-the-ear microphones and may not have enough memory or data transfer rates to test real-time strategies, nor may they be portable enough for take home studies. Therefore, with these research interfaces, the benefit of ITD cues have not been evaluated in free field listening settings with processor microphones due to the lack of bilaterally-synchronized research processors with access to live microphones. Instead, it has been evaluated in a direct stimulation setup, where processor microphones are bypassed, and the stimulation is sent directly to participant’s implants (Best et al., 2011; Egger et al., 2014; Thakkar et al., 2018, 2023). In most cases, envelope information was eliminated so that there were no ILD cues, which BiCI users rely on in the free field when their processors lack ITD cues. The CCi-MOBILE research platform was developed at the University of Texas at Dallas (Ghosh et al., 2022) to facilitate the development and real-time validation of new coding strategies with bilateral synchronization (Azadpour et al., 2025). More importantly, the CCi-MOBILE has the ability to bilaterally synchronize the incoming microphone inputs that drive the output of a coding strategy. By using the CCi-MOBILE, this work demonstrates the first study evaluating the benefit of ITD cues encoded by synchronized low-rate stimulation in a free field sound localization experiment.

In this study, we investigated the “mixed rates” strategy using the CCi-MOBILE for real-time, free field horizontal sound localization. Churchill et al. (2014) established that ITD sensitivity could be partially restored to BiCI listeners while maintaining speech intelligibility by extracting acoustic fine structure timing and using that information for pulse timing in four low-frequency channels. Later studies clarified that ITD sensitivity can be successfully measured with as few as a single low-rate channel (Thakkar et al., 2018, 2023). With these studies in mind, we developed a real-time-capable implementation of the mixed rates strategy that estimates the acoustic ITD every 8 ms and directly encodes this cue in the timing of select low-rate channels (Dennison et al., 2024). However, there has been great individual variability in the outcomes between participants as quantified by direct-stimulation lateralization measurements, which can be explained by the fact that the sensitivity to ITDs can vary along the electrode array (Laback et al., 2015). In other words, the locations with the “best” (lowest) thresholds for detecting ITDs could be different between individuals. Our most recent work investigated the personalization of the mixed rates strategy by deliberately directing low-rate stimulation to an electrode pair with the best ITD sensitivity (Borjigin et al., 2024). This patient-specific strategy yielded better results than the test condition that assigns low-rate stimulation to the electrode with the poorest ITD sensitivity and the clinical-like strategy without any low-rate stimulation. This personalization also ensured that only one pair of electrodes was used for low-rate stimulation while the remaining channels kept high-rate stimulation for speech comprehension. The success of this personalized mixed rates strategy in optimizing ITD cue encoding represents a significant step forward toward a precision medicine approach in the programming of BiCIs.

Here, we present the first free field evaluation of the mixed rates strategy using CCi-MOBILE in a horizontal sound localization task, where ILD and ITD cues coexist and naturally vary with each participant’s head cues. In addition to synchronizing microphone inputs, the CCi-MOBILE also allows the measurement of the binaural cues delivered to the user with their own head cues. The free field evaluation of these mixed rates strategies is an important step toward bringing these novel strategies into clinical application. We hypothesized that good ITD sensitivity is a necessary requirement for CI listeners to benefit from mixed rates strategies in the free field. If so, listeners with low thresholds (i.e., good sensitivity to ITDs with single electrode pairs) will show less localization error when using a mixed rates strategy compared to all-high or clinical strategies, which do not deliver synchronized ITDs at low rates.

## 2 Materials and methods

### 2.1 Participants

Fourteen bilateral cochlear implant (BiCI) users participated in this study. All participants were users of Cochlear Ltd. devices (Sydney, Australia), as the research processor employed, CCi-MOBILE (see below), was only compatible with the Cochlear Nucleus24 implant at the time of testing. Participants were selected based on their demonstrated sensitivity to interaural time differences (ITDs) with at least one electrode pair, as determined by prior studies conducted in our lab (Thakkar et al., 2020). Participants received a stipend for their time, along with reimbursement for all travel-related expenses. Demographic information is provided in Table 1. All procedures adhered to National Institutes of Health guidelines and best practices for direct stimulation studies (Litovsky et al., 2017), and were approved by the Institutional Review Board of Health Sciences at the University of Wisconsin-Madison.

**Table 1:** Demographic information of BiCI listeners.

ID	Sex	Age at testing (years)	Age at hearing loss (L, R)	Age at implantation (L, R)	Experience with BiCIs (years)	Bilateral hearing loss before BiCIs (years)	Etiology (L, R)
IAJ	Female	78	5, 5	51, 58	20	53	H, H
IAU	Male	74	3, 3	50, 56	18	53	H, H
IBF	Female	72	38, 38	56, 54	16	18	H, H
IBL	Female	77	12, 12	54, 59	18	47	U, U
IBO	Female	58	23, 23	45, 42	13	22	O, O
IBY	Female	60	41, 41	43, 48	12	7	U, U
ICD	Female	65	3, 3	50, 44	15	47	EVA, EVA
ICI	Female	65	31, 31	50, 51	14	20	U, U
ICM	Female	70	23, 23	57, 58	12	35	U, U
ICP	Male	61	4, 4	46, 49	12	45	U, U
IDA	Female	57	8, 8	47, 46	10	39	U, U
IDL	Female	69	33, 33	62, 61	7	29	U, U
IDM	Female	46	5, 5	33, 35	11	30	U, U
IDO	Male	52	46, 38	46, 43	6	0	SI, ISL

EVA, enlarged acoustic aqueduct; H, hereditary; ISL, idiopathic sudden loss; O, otosclerosis; SI, skull injury; U, unknown. Bilateral hearing loss was calculated by subtracting the age at hearing loss (later ear) from the age at implantation (second implant).

### 2.2 Experimental design and statistical analyses

#### 2.2.1 Experiment conditions

**Figure 1:**
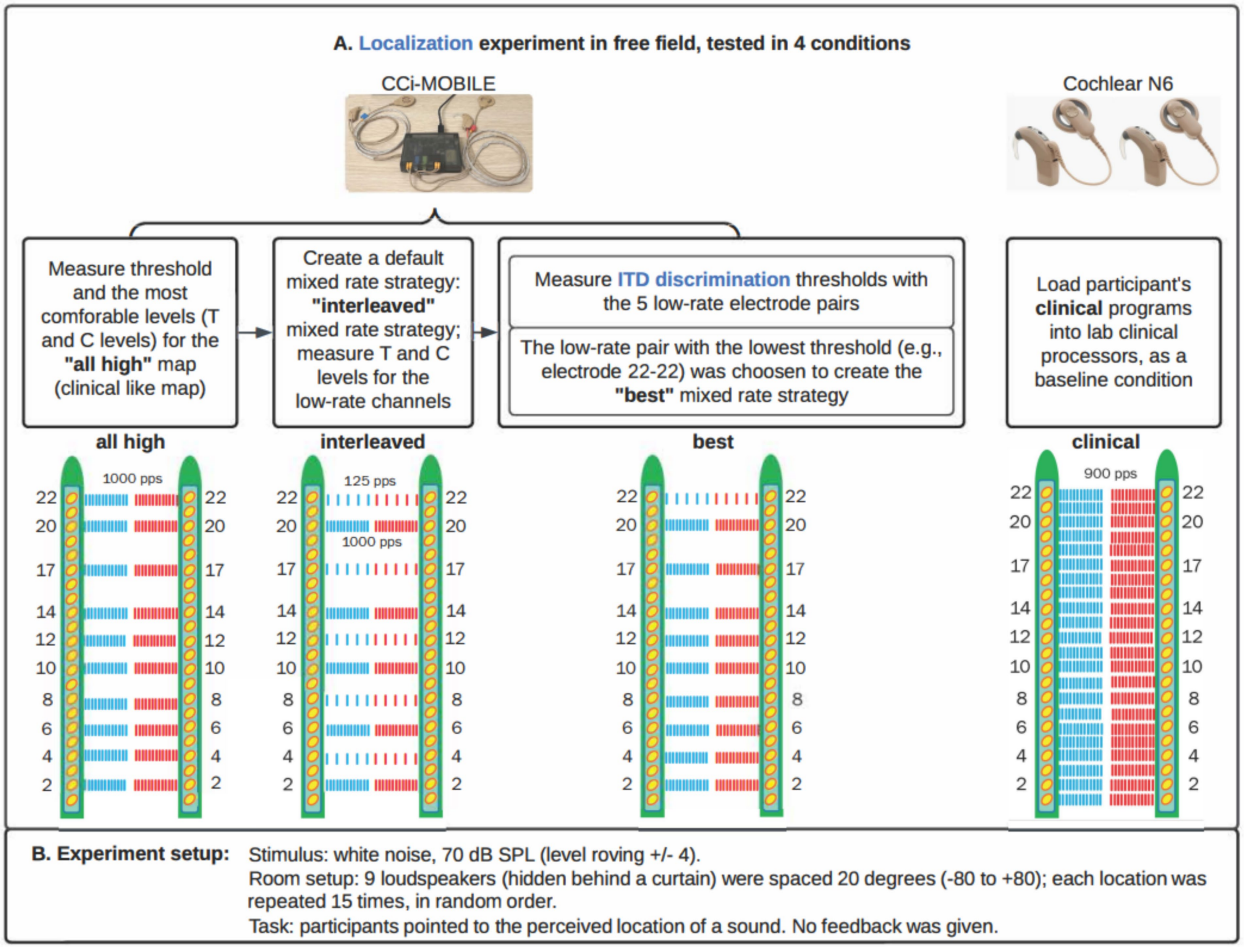
Stimulation strategies/conditions. **(A)** Four stimulation strategies/conditions for this study. Each participant had 10 electrodes activated in each ear for all three strategies implemented on CCi-MOBILE. **(B)** Experiment details.

In this study, we compared four stimulation conditions. Three were implemented with the CCi-MOBILE: a version of Continuous Interleaved Sampling (CIS) labeled “All-high” (i.e., high-rate stimulation at all stimulating electrodes), “Best” mixed rates where low rates were only provided to a single pair of electrodes with the lowest ITD threshold, and “Interleaved” mixed rates where every other electrode had low rate stimulation. Ten electrode pairs were stimulated for all three strategies using CCi-MOBILE. By default, electrodes 2, 4, 6, 8, 10, 12, 14, 17, 20, and 22 were selected on both sides. If any of these default electrodes were deactivated in a participant’s clinical map, the selection was adjusted accordingly. For the Interleaved Mixed Rates strategy, electrodes 4, 8, 12, 17, and 22 were designated as low-rate channels by default. For the Best mixed rate strategy, low rate stimulation was provided on the electrode pair with the lowest ITD threshold from the set of low rate electrodes (4, 8, 12, 17, 22). The fourth condition was clinical condition: each participant’s every day (typically 22 pairs of electrodes) clinical strategy run on the clinical processors, which were not synchronized between the ears. Figure 1A visually summarizes each stimulation condition. Table 2 shows frequency allocation for each electrode.

**Table 2:** Standard frequency allocation table (FAT).

Channel	Electrode	Cutoff frequency (Hz)		
		Lower	Center	Upper
1	2	6,063	6,501	6,938
2	4	4,688	5,001	5,313
3	6	3,563	3,813	4,063
4	8	2,688	2,876	3,063
5	10	2063	2,188	2,313
6	12	1,563	1,688	1813
7	14	1,188	1,251	1,313
8	17	813	876	938
9	20	438	501	563
10	22	188	251	313

Some participants used adjusted electrode numbers but had identical frequency allocations. Cochlear device has 22 intra-cochlear and 2 ground electrodes.

The algorithm for the mixed rate strategies, as adapted from Dennison et al. (2024), consisted of the following steps:

1 Read 8 ms of stereo audio. 8 ms is set by the firmware. Input is synchronously recorded with two microphones at a sampling frequency of 16,000 Hz in 8 ms frames. The buffered frames are processed as overlapping blocks of 128 samples, with a hop size of 1 ms and 7 ms of overlap in each block. A 128-point Hann window is applied to each block. The Hann window is calculated as: *w*(*n*) = 0.5–0.5 cos(πn/2), for *n* = 0 to 127.
2 Extract envelope for 10 channels. In each ear separately, a 128-point Fast Fourier Transform (FFT) is then applied to each block. Only the first 65 bins are retained, discarding bins for negative frequencies. The complex values in the transformed frame are multiplied by their complex conjugates to estimate the power in each frequency bin. A frequency-weighted scaling is applied based on how many channels are active. The frequency bins are consolidated into 10 frequency channels with center frequencies matched to the standard Frequency Allocation Table. The square root of each entry in this matrix is then calculated to provide an estimate of the channel energy. For low-rate channels, the mean magnitude in the entire block is used as the envelope.
3 Estimate ITD based on cross-correlation. The left and right frames in a block are compared with a cross-correlation in the time domain, and the delay that maximizes the cross-correlation is used as the ITD for the entire 8 ms of stimulation. The delay is rounded up to the nearest multiple of 100 μs. Multiples of 100 μs were used because the overall stimulation rate of the mixed rate strategy is 10,000 pps, and the highest possible resolution pulse timing without needing to implement a pulse collision avoidance algorithm is the reciprocal of this rate, which is 100 μs.
4 Apply estimated ITD and envelope to low-rate pulse. Depending on the delay estimated in the ITD estimation step, either the left or right pulses are delayed to encode the ITD. Because at the time of study, Cochlear devices could only stimulate one electrode at a time in each ear, any high-rate pulses that overlap in time with the low-rate pulses are removed. If there is an ITD of 0 μs, pulses of the two implants will be simultaneously scheduled in the low-rate channels. The amplitude of the low-rate pulses is the average energy over the entire 8 ms frame for that channel.
5 Map to threshold and comfortable levels. Dynamic range compression is achieved using a logarithmic function to map the normalized amplitude levels to the current levels for each channel based on the Threshold (T) and Comfortable (C) levels in each patient’s clinical MAPs, which map to the softest and loudest sounds that a patient could hear.

**Figure 2:**
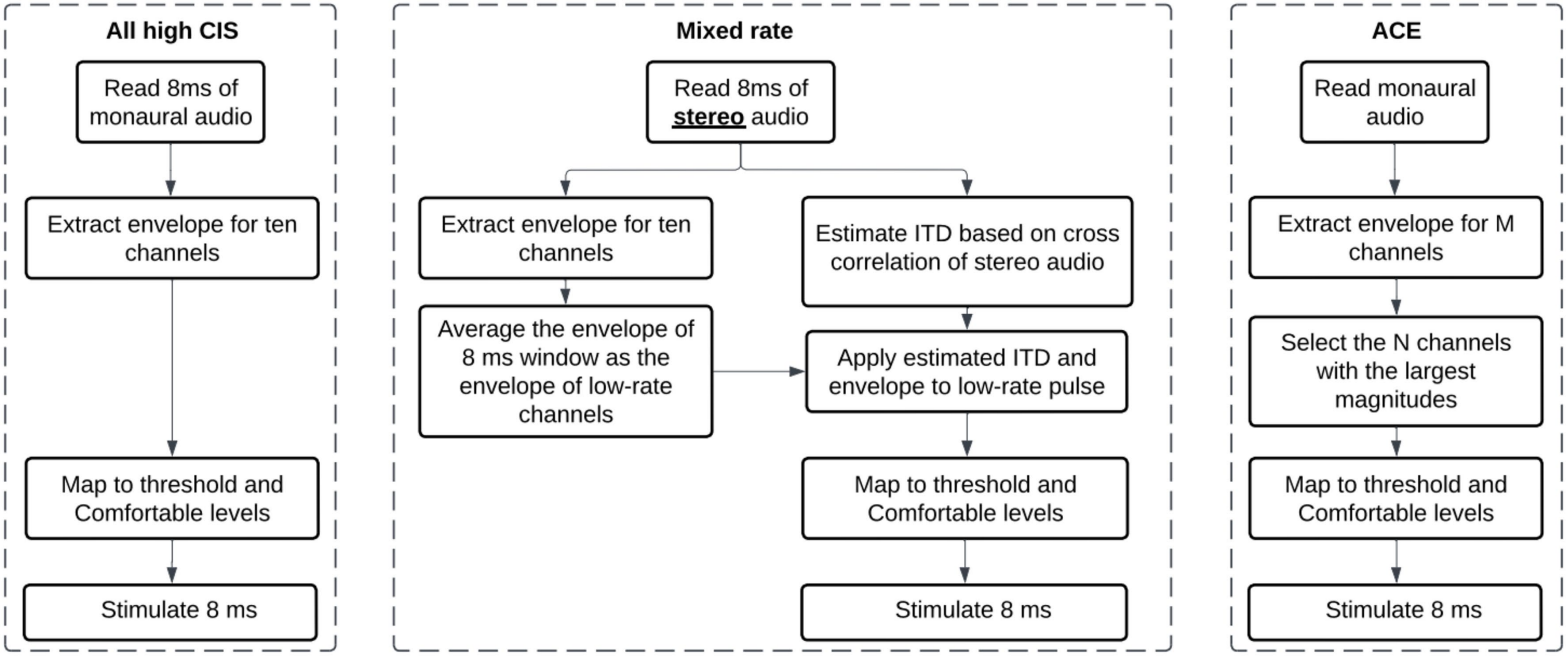
Flow diagrams explaining the three types of stimulation strategies compared in the study. “Interleaved” and “Best” strategies are Mixed rate strategies, only varying in which channel is a low-rate channel.

We implemented each processing condition on the CCi-MOBILE using custom MATLAB software (R2022b) running on a Microsoft Surface Microsoft, Redmond WA, United States; Intel(R) Core (TM) i7-1065G7 CPU @ 1.30GHz 1.50GHz, 16 GB RAM with Windows 10 operating system. Within a single participant, the same set of 10 electrodes was activated for both ears for the three strategies tested with CCi-MOBILE. The All-high strategy used high-rate stimulation of 1,000 pulses per second at all 10 electrode pairs. In the Interleaved mixed rates strategy, every other electrode pair received low-rate stimulation of 125 pps. The interleaved pattern was selected for this study so that ITD information would be provided at different locations all along the electrode array. ITDs were encoded in the low-rate channels but not explicitly encoded in the timing of pulses in the high-rate channels. We judged that it was highly unlikely that these arbitrary ITDs would compromise the ITDs provided on low-rate channels because sensitivity to ITDs would be poor at 1,000 pps due to the rate limitations BiCI users experience (Anderson et al., 2019; Carlyon et al., 2025; Kan and Litovsky, 2015; Laback et al., 2007; van Hoesel and Tyler, 2003). The Best mixed rates strategy had a single pair of electrodes stimulated at low rate (125 pps), while the remaining nine pairs of electrodes received high-rate stimulation of 1,000 pps. Low-rate stimulation was sent to the single electrode pair with the lowest (i.e., the best) ITD discrimination threshold. ITD discrimination was measured at all five pairs of low-rate electrodes in the Interleaved mixed rates strategy to determine which pair of electrodes leads to the best ITD sensitivity. The clinical strategy contained the same set of electrodes as in the participant’s everyday strategy and was run on a pair of clinical processors. The three strategies implemented on CCi-MOBILE are similar to Continuous Interleaved Stimulation (CIS) but with synchronization between ears. The clinical strategy run on clinical processors adopts the Advanced Combination Encoder (ACE) strategy. Automatic gain control (AGC) was used for the clinical strategy, but not for strategies running on CCi-MOBILE, so that AGC would not interact with the ITD coding. Figure 2 shows more details on the processing steps of the stimulation strategies.

#### 2.2.2 Stimuli, procedure, and equipment for localization

##### 2.2.2.1 Loudness mapping

We first measured the threshold (T) and most comfortable (C) loudness levels on each electrode with low- and high-rate stimulation. Note that T and C levels were only remeasured for the ten electrodes selected for stimulation strategies. Mapping stimuli consisted of 300 ms constant-amplitude pulse trains delivered at either 125 or 1000 pulses per second, depending on whether the channel was low- or high-rate. The pulse widths corresponded to the clinical setting of each participant. The interphase gap duration was set to 8 us. Three distinct maps were created for this study, all using the same set of ten electrodes. The default electrode selection for both sides included electrodes 2, 4, 6, 8, 10, 12, 14, 17, 20, and 22. The electrode selection was modified slightly if any of the default electrodes were deactivated in the participant’s clinical map. For the Interleaved mixed rates strategy, we assigned electrodes 4, 8, 12, 17, 22 as low-rate channels by default (see Figure 1A, interleaved condition). Following measurement of T and C levels for all electrodes in each ear, loudness was balanced across electrodes. C levels were swept across multiple electrodes, initially in groups of three adjacent electrodes with one overlapping electrode between neighboring groups, and subsequently in groups of five adjacent electrodes. Loudness was also balanced between the ears by stimulating each pair of electrodes simultaneously, making sure that the stimulation resulted in a centered intracranial percept.

##### 2.2.2.2 ITD discrimination

We tested ITD discrimination with a two-interval, two-alternative forced choice (2AFC) task to determine the “best” electrode pair. The interstimulus interval was 300 ms. The ITD magnitude was identical in both intervals, but the polarity was reversed. Each interval contained a 300 ms constant-amplitude pulse train at 125 pps, delivered with an interaural delay. One hundred twenty-five pps was chosen because we used 125 pps stimulation for low-rate channels in the mixed rates strategies. Participants used buttons on a graphical user interface to indicate whether the second interval was to the “Left” or the “Right” of the first interval. We used the constant stimuli method to measure the just noticeable differences (JNDs) or discrimination thresholds, with a default selection of ITDs: 50, 100, 200, 400, and 800 μs. These ITD values were chosen to range from a value that was sub-threshold to most listeners (50 μs) to a value that was larger than most human head sizes (800 μs), with a logarithmic spacing. If needed, we added additional ITDs below 50 μs and/or above 800 μs to get a complete psychometric function. To determine whether additional ITDs were needed, we broke down the data collection into many runs and plotted the data after each run for monitoring purposes. We estimated JNDs as the 75% correct point along the psychometric curve for 2AFC. Data were fit using the psignift MATLAB package developed based on Kuss et al. (2005). We presented each ITD 40 times to each electrode pair, with half of the trials leading to the left first and half of the trials leading to the right first. We measured ITD JND at one electrode pair at a time. Note that we adjusted the stimulation levels on two sides to ensure a centered auditory image (that is, C levels were balanced between ears; see the “Loudness mapping” section above), or in other words, we kept ILD fixed at zero during the ITD discrimination task. We provided an initial feedback training in the beginning but turned it off during the formal data collection.

##### 2.2.2.3 Localization

Localization stimuli were one-second-long burst of broad-band white noise and presented at an average level of 70 dB sound pressure level (SPL) (Figure 1B). White noise has been used in localization experiments with BiCI listeners in previous studies (Moua et al., 2019). In each presentation, we roved the sound level in the range of ±4 dB to discourage the use of monaural loudness cues, which may distinguish loudspeakers by perceived loudness based on the overall level in a single ear. The stimuli were delivered from nine loudspeakers (Center/Surround IV; Cambridge SoundWorks) arranged in a semicircular arc, positioned approximately 1.2 meters from the participant’s head. We spaced the speakers at 20-degree intervals, covering an azimuth range of ±80 degrees. We adjusted the listener’s seat height to align the listener’s head with the loudspeakers at zero-degree elevation. The loudspeakers were concealed behind a dark, acoustically transparent curtain. We presented the stimuli using a Tucker-Davis Technologies System3, comprising units RP2.1, HB7, and PA5 (digital processor, amplifier, and attenuator, respectively). We ran signal processing and presentation on a desktop computer with custom-written MATLAB software (R2016b). We carried out the tests in a single-walled sound attenuating booth (Industrial Acoustics Company, Inc.) measuring 2.90 × 2.74 × 2.44 meters, with sound attenuating foam attached to some of the walls to minimize reflections.Before testing, we played the noise burst from a loudspeaker positioned directly in front of the participant’s head without level roving. If the perceived location of the sound was skewed to one side, the volume on the sound processor was adjusted until the participant perceived the sound coming from the loudspeaker in front. This adjustment to perceive the sound as coming from the front loudspeaker was performed for all four stimulation strategies. The overall loudness was also adjusted for all four strategies to ensure that all strategies resulted in approximately the same loudness perception. Volume adjustments were documented for each participant. We provided familiarization sessions before testing to help participants understand the task. During familiarization, the use of the graphical user interface and the task was demonstrated and explained. Participants were instructed to face forward and keep their heads as still as possible before starting each test block. In each trial, stimuli were presented from one of the nine loudspeakers. The task of the participant was to identify the location of the target loudspeaker. Participants initiated each stimulus presentation using a graphical user interface implemented in MATLAB on a touchscreen located directly in front of them. Following stimulus presentation, participants could respond by pointing a remote with laser light toward the desired location in the horizontal place and pressing a button on the remote to confirm their response. We determined the exact location and orientation of the remote in space by four infrared motion-capturing cameras (OptiTrack, Natural Point Inc., Corvallis, OR, United States) mounted on the ceiling of the sound booth. The position and orientation of the laser pointer was inferred from the camera data and projected onto an azimuthal angle along the loudspeaker array (Warnecke et al., 2020). This angle was used as the response angle. Participants were unable to repeat stimulus presentations. No feedback was provided after each trial. The test was completed in four blocks: each was tested with a different stimulation strategy and contained 135 trials (15 repetitions x 9 loudspeakers). Testing took about 30 min to complete. The order of the blocks was counterbalanced among the participants using the Latin-square randomization procedure.

##### 2.2.2.4 Devices

We performed the loudness mapping and ITD discrimination tasks using the Nucleus Implant Communicator (NIC) (RF GeneratorXS, Cochlear, Sydney, NSW, Australia). Custom written MATLAB software (Mathworks, Natick, MA) was used to create the testing interface, which generated and sent the stimuli directly to the participant’s implants. The localization task used the CCi-MOBILE. CCi-MOBILE allows simultaneous processing and stimulation of a pair of Cochlear internal implants via the Windows Surface device mentioned above. Note that this is just for running the CCi-MOBILE, a different computer than the one used for stimulus presentation. CCi-MOBILE is bilaterally synchronized, which means that a single clock is used to drive two internal devices simultaneously (see Dennison et al. (2022) for a discussion of synchronized processors). CCi-MOBILE is also capable of taking in microphone inputs in the free field, just like clinical processors, but with synchronization. This important feature makes it possible to test custom research strategies (e.g., mixed rates strategy) in the free field with synchronization.

#### 2.2.3 Measurement and computation of acoustic and electric binaural cues

**Figure 3:**
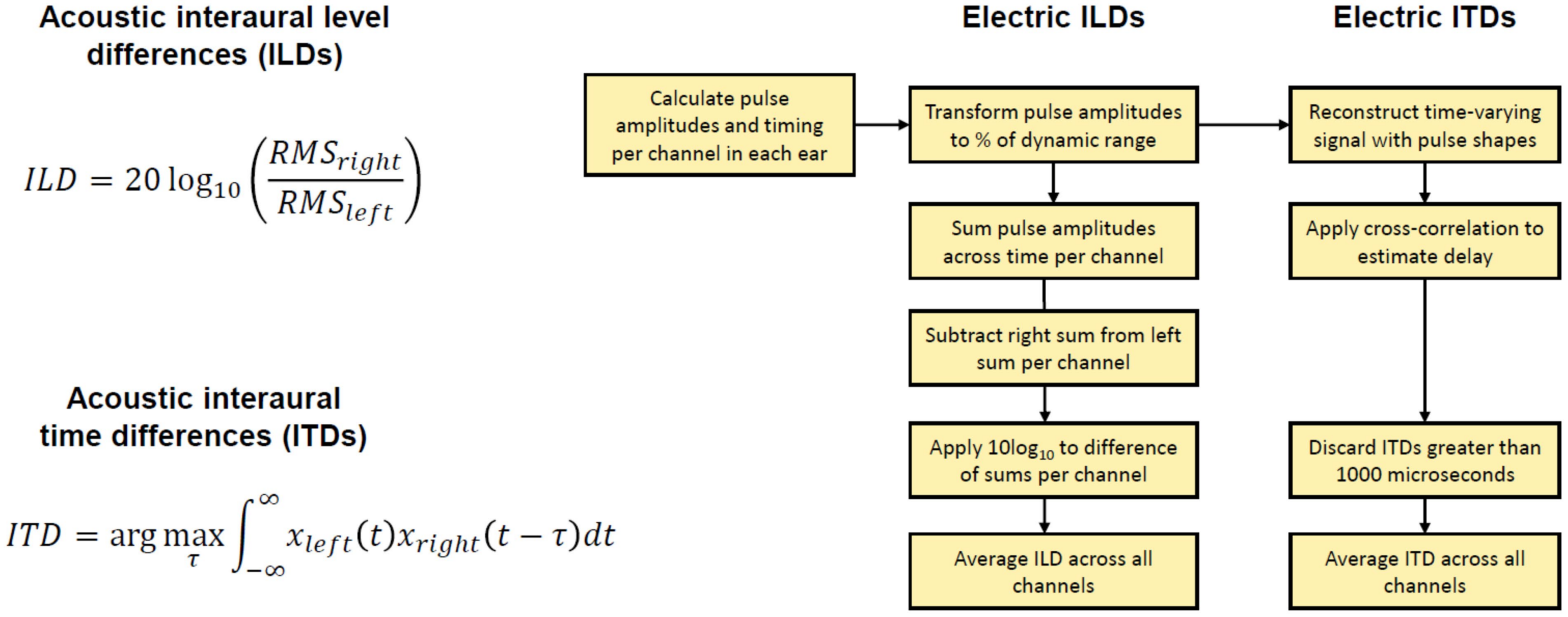
Explanation of binaural cue analysis. Left: Acoustic binaural cues are calculated with provided equations. Right: Electric binaural cues are calculated following the steps in the block diagram. RMS, root mean square of signal.

After the localization task, we also recorded the acoustic stimulus from each loudspeaker several times, without level roving. We collected the recordings using CCi-MOBILE, with two processor microphones placed on the participant’s two ears (just as in the localization experiment). Therefore, these recordings implicitly capture the head-related transfer functions (HRTFs) of each participant wearing the CCi-MOBILE behind-the-ear (BTE) microphones used during the experiment. During the recordings, the participant was instructed to sit still, facing the center loudspeaker. They were asked not to respond to stimuli during the recording. The audio recordings were collected to analyze the presence of ITD and ILD cues in the acoustic stimuli at the microphone input level. The audio recordings were also further processed by the participant’s four stimulation strategies and transformed into the electrical stimulation pattern —electrodogram. The presence of ILD and ITD cues was analyzed by comparing electrical pulses in the electrodograms on two sides. Figure 3 summarizes the analysis described below.

##### 2.2.3.1 Acoustic binaural cue analysis

The binaural recordings were saved in wav format at a sample rate of 16000 Hz. The recordings were completed in a single session per each participant. We calculated the broadband binaural cues available in these recordings. We avoided any assumptions about the frequency weighting of ILDs or ITDs. ILDs were calculated with the equation: ILD(*θ*) = 20 log_10_(*x_right,RMS_*(*θ*)*/x_left,RMS_*(*θ*)), where *θ* is the position of the loudspeaker in angle and *x_left,RMS_* and *x_right,RMS_* are the root mean square (RMS) of the left and right waveforms corresponding to the loudspeaker, respectively. ITDs were calculated as the delay that maximizes the cross-correlation between the left and right signals. If the delay exceeded 1,500 μs, a delay of 0 μs was used. A negative ITD indicates an ITD pointing toward the left, while a positive ITD indicates an ITD pointing toward the right. The cue value was then averaged over the repetitions for each loudspeaker location.

##### 2.2.3.2 Electric binaural cues

There are no uniformly agreed methods to estimate binaural cues from electrical stimulation, although some authors offer thorough examples of how to estimate cues from electrical stimulation (Gray et al., 2021; Kan et al., 2018). In our study, to estimate the binaural cues, we processed recordings from the CCi-MOBILE microphones with the strategies and settings used in the experiment for each participant. We calculated individualized electric binaural cues for each stimulus at each loudspeaker location by analyzing the output of each strategy. The outputs of each strategy were vectors of pulse amplitudes and timings for each electrode in both ears. In the localization experiment, this information was transmitted by the CCi-MOBILE coils to the internal devices. However, when processing offline, these outputs can be saved and analyzed as we did here. To estimate ITDs, electrical pulse trains were reconstructed for each active channel (10 for the mixed rates or all-high strategy, and up to 22 for the clinical strategy). The pulse trains consisted of biphasic pulses determined by participant-specific parameters including the pulse duration, inter-pulse gap, and stimulation amplitude. Then, we estimated the electrical ITDs as the delay that maximized the cross-correlation between the left and right pulse trains. ITDs greater than 1,000 us were assigned as NaNs, as the mixed rates coding strategy cannot encode ITDs larger than that value. The final ITD estimate was averaged across all 10 active channels for the CCi-MOBILE conditions or all channels in the Clinical strategy where channel numbers matched across ears. To estimate ILDs, the amplitudes of each electric pulse were first transformed into a percentage of the dynamic range according to the patient maps. To achieve this, the threshold current level was subtracted from the pulse current level, and this difference was then divided by the difference between the maximum current level and the threshold current level (i.e., dynamic range of a particular channel). This conversion to DR was done pulse by pulse. This transformation was meant to accommodate different current levels and dynamic ranges between the electrodes and ears. For example, after conversion to dynamic range, pulses could have a percentage between 0 and 100. ILDs could then be calculated across the ears for each channel in each mixed rates map as decibel difference in energy between two ears (log 10 of RMS power in the right ear over that in the left ear). The final ILD estimate was averaged across all 10 active channels for the CCi-MOBILE conditions or all channels in the Clinical (ACE) strategy where channel numbers matched across ears. ACE was simulated for the Clinical condition using the CCi-MOBILE code.

#### 2.2.4 Statistical analyses

For analyzing the localization data, the root mean square (RMS) of the localization error was calculated at each speaker location, where the error is the difference between the actual response location and the speaker location. We then extracted a single metric from each participant by calculating the RMS of the localization errors from all the speaker locations. We performed statistical analysis with RStudio running R (version 4.3.1). To test whether BiCI listeners would show better localization performance with the Best and Interleaved mixed rates strategies than with the All-high strategy and unsynchronized clinical strategy, we used a linear mixed effects model (lme4 package, version 1.1.31) with localization performance (RMS error) as dependent factor and stimulation strategy as independent factor, with random effects to account for variability associated with participants: *model* = *lmer*(*lateralization error* ∼ *stimulation strategy* + 1|*participant*). We used the anova function (car package, version 3.1-2) to calculate the Type III sequential sum of squares, assessing the predictive contributions of independent factors and their interactions in the linear mixed-effects model. Residual normality was visually evaluated using Q–Q plots and confirmed with Shapiro-Wilk tests. The homogeneity of the variance was assessed using Levene’s test on the residuals of the model. Post hoc comparisons were performed using estimated marginal means analysis via the emmeans package (v1.8.9). We conducted Pearson’s correlational analysis between ITD JNDs (both the best and worst JNDs within each participant) and the participant’s localization performance. Due to a violation of the assumption of normality, ITD JNDs (range: 30.2–1871.7 μs) were first logarithmic transformed.

## 3 Results

### 3.1 ITD discrimination for customizing mixed rates strategy

**Figure 4:**
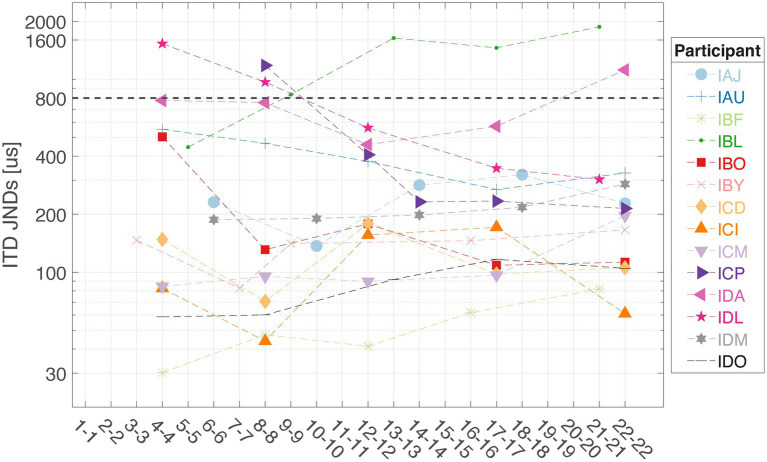
Interaural time difference JNDs were measured at five locations along the electrode array for each listener in this study (*n* = 14). The dashed line at 800 μs represents the approximate upper limit of ecologically relevant ITDs for humans.

Figure 4 shows the ITD JNDs measured at five locations along the electrode array for each individual. This step identifies the electrode pair with the lowest ITD JND for 100 pps pulse trains delivered through direct stimulation, which will then be assigned for low-rate stimulation in the mixed rates strategy. The customized mixed rates strategy contains a single low-rate channel with the other remaining nine pairs of electrodes receiving standard high-rate stimulation and is referred to as “Best” mixed rates strategy (see Figure 1 for an example stimulation pattern). Multiple factors may contribute to variability in ITD sensitivity across the electrode array. A key factor is interaural asymmetry, such as unequal neural survival at corresponding electrodes or differences in insertion depth between the two ears (Kan et al., 2013a; Kan et al., 2013b; Kan and Litovsky, 2015).

### 3.2 Localization with custom mixed rates strategy

**Figure 5:**
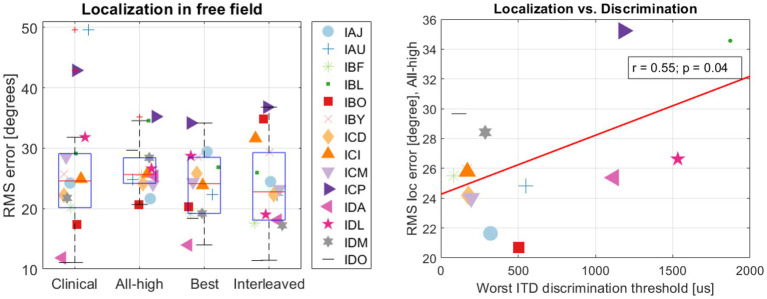
(Left) summary of RMS errors across strategies and (right) the correlation between localization and discrimination.

**Figure 6:**
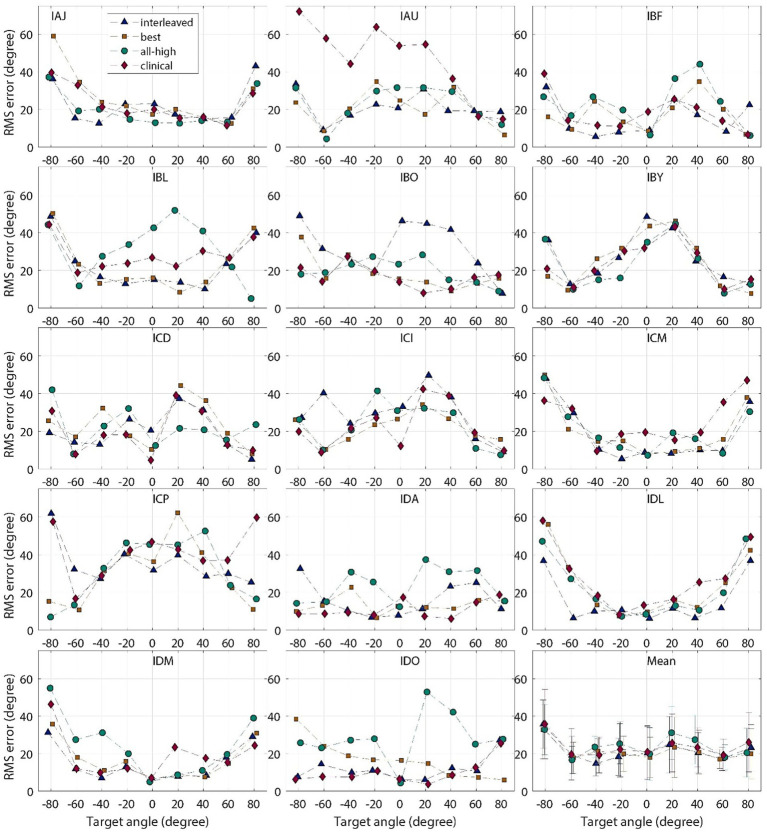
Individual and group mean RMS localization errors plotted for each target location, for “all-high” “best,” “interleaved,” strategies tested with CCi-MOBILE and clinical strategy with clinical processors.

**Figure 7:**
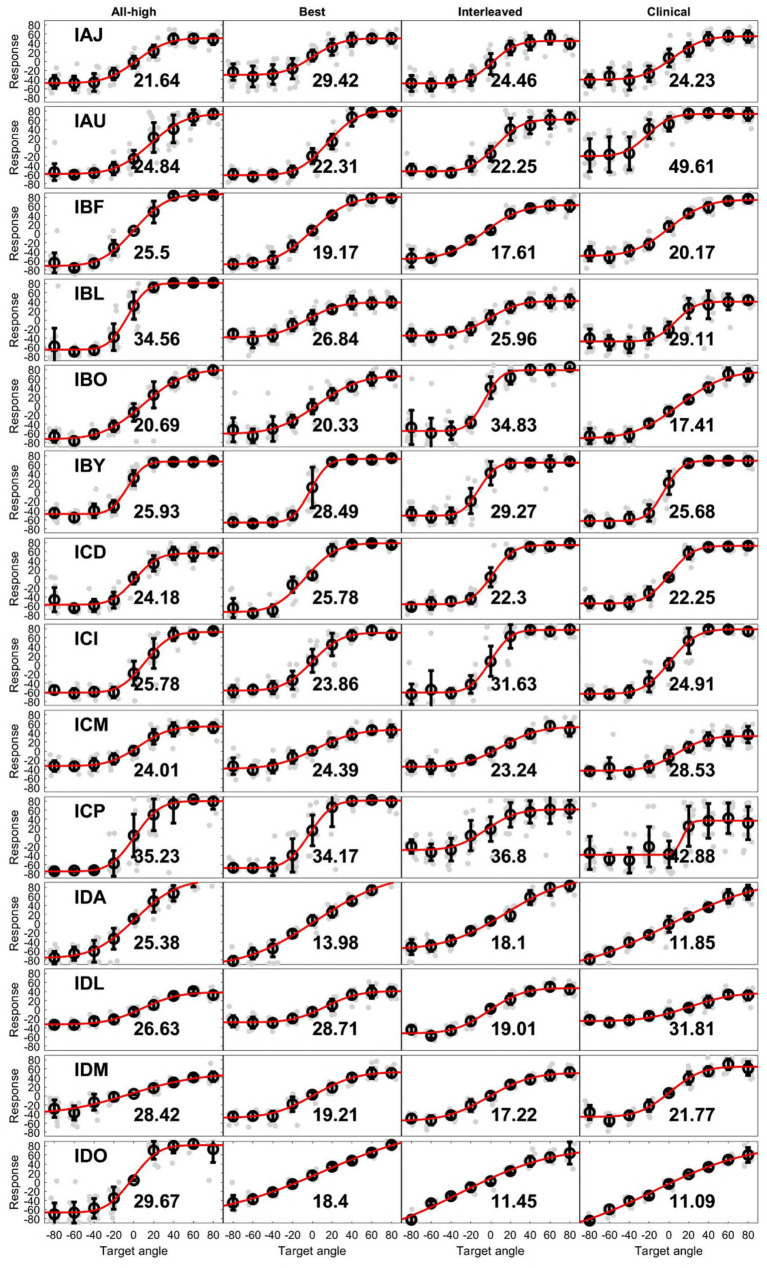
Unprocessed localization data. Each row contains data with 4 strategies from an individual. Light gray dots are individual responses corresponding loudspeaker locations (target angle). The circle at each target angle is the mean of the individual dots while the error bar is 1 standard deviation. The red line is the fit of means at all target angles. The number in each plot is the RMS error from that condition.

On population level, BiCI users did not benefit from the Best or Interleaved mixed rates strategy in localization, compared to the All-high synchronized strategy and the unsynchronized clinical strategies [see Figure 5 (left) for the summary of root mean square (RMS) errors across four stimulation strategies tested; see Figure 6 for RMS errors at each individual speaker location for each strategy within each individual]. Note that some individuals did benefit from the Best and/or Interleaved mixed rate strategies, which is detailed in the Discussion section. Figure 7 shows the localization responses of each individual across all trials for each strategy. Figure 5 (right) shows a positive correlation between the ITD discrimination thresholds in the direct stimulation setup and the RMS localization error in the free field (*r* = 0.55, *p* = 0.04). The positive relationship means that a higher (worse) localization error was correlated with higher (worse) ITD thresholds.

### 3.3 Measurements of binaural cues provided by processing strategies

**Figure 8:**
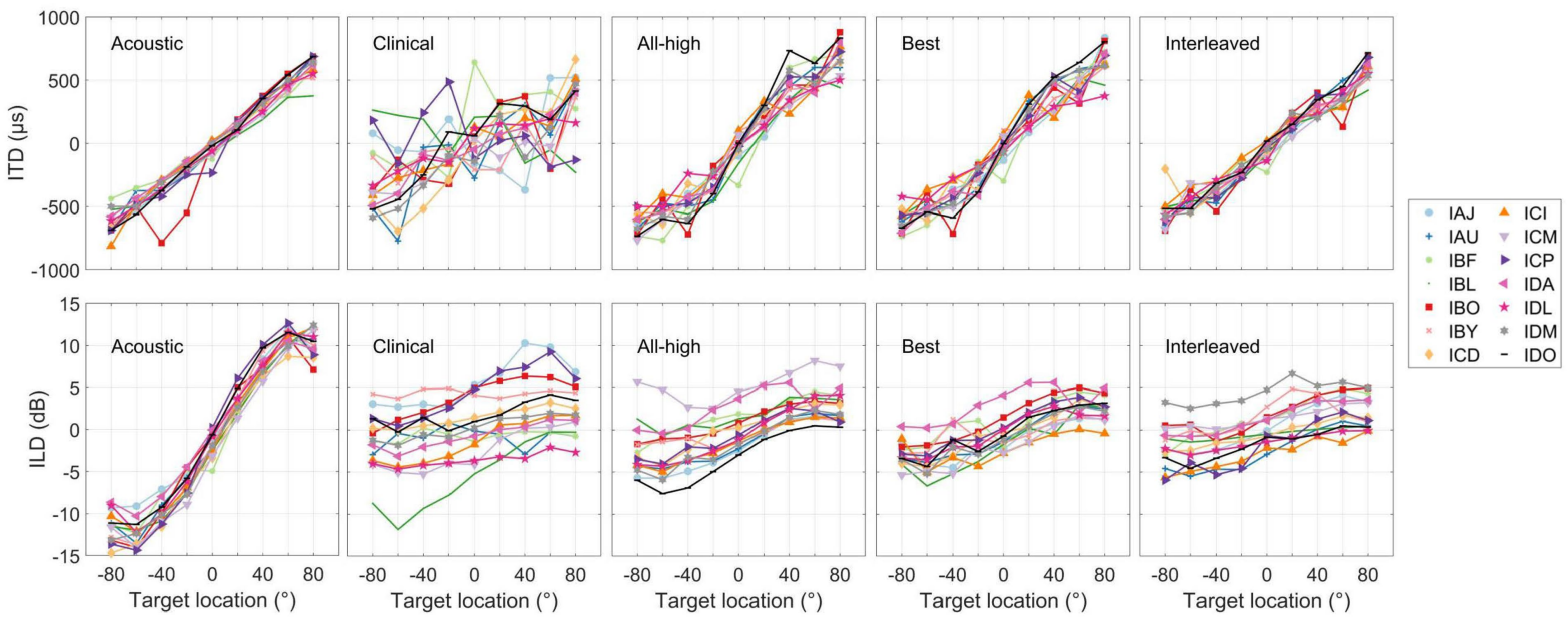
Summary of interaural level and time differences measured for the heads of individual participants (see panels labeled as Acoustic) and estimated from stimulation output of each stimulation strategy. Each curve represents the mean cue per person per strategy.

Figure 8 (“Acoustic” panels) summarizes the binaural cues recorded from the BTE microphones of the CCi- MOBILE. Across participants, the cues in the acoustic signals were remarkably consistent, particularly for the ITDs. The ITDs from the acoustic recordings were linear as a function of loudspeaker location, while the ILDs from the acoustic recordings were also similar across participants but had non-monotonic shape. This suggests that the participants received consistent binaural cues (diagonal one-to-one mapping between the estimated cues and actual speaker locations) that are comparable to typical acoustic hearing and that there were no large differences in acoustic inputs to the CCi-MOBILE that could drastically influence the extraction and encoding of binaural cues in electrical stimulation. The panels labeled by the processing strategies in Figure 8 summarize the electrical binaural cues estimated from the stimulation outputs of the CCi-MOBILE with each stimulation strategy for each participant. Electric ITDs were more variable than acoustic ITDs but still were linear, except for clinical condition. Electric ILDs varied much more than ITDs and were compressed compared to acoustic ILDs. However, the electrical ILDs are still relatively linear in all four conditions.

**Figure 9:**
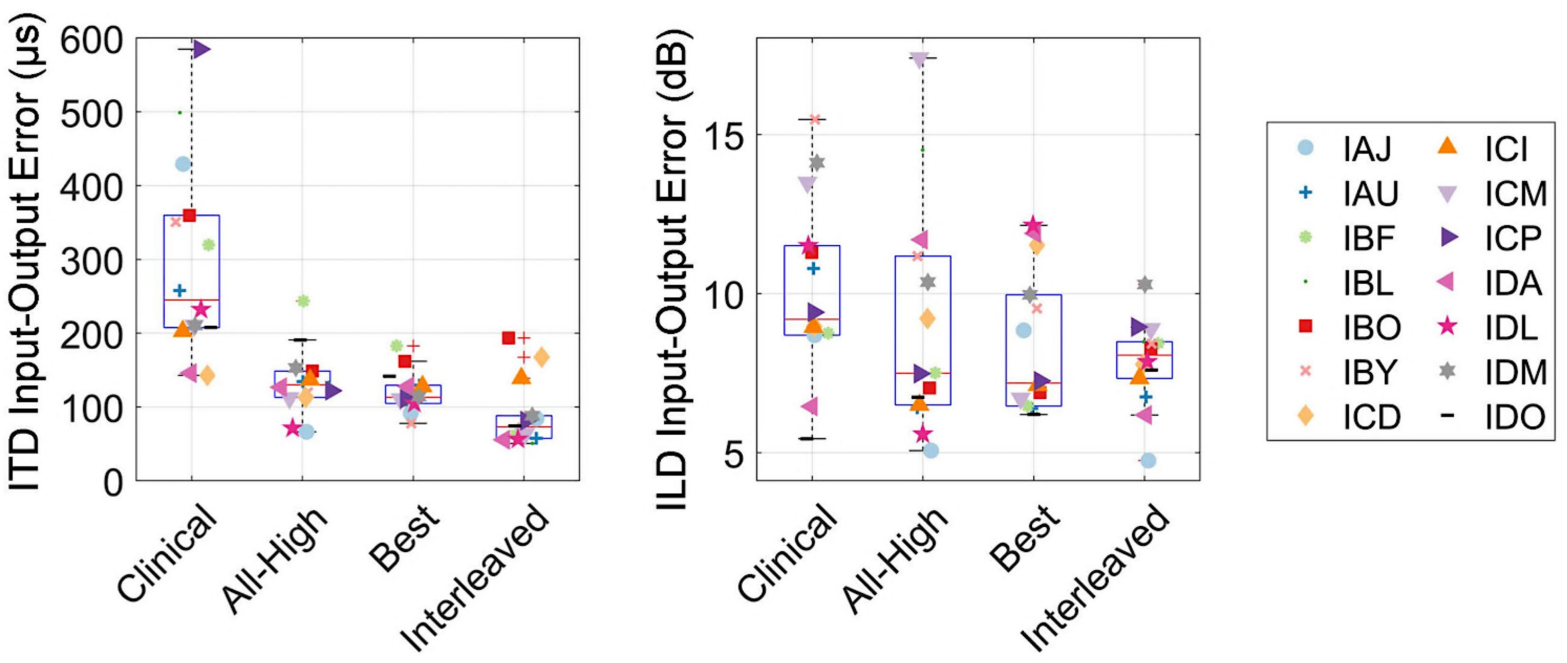
Input–Output errors between acoustic and electric ITD and ILD cues, measured as the RMS error. RMS errors between the acoustic and electric ITD and ILD cues.

Figure 9 shows the accuracy between the binaural cues calculated in the acoustic recordings and the electrical signals. The overall RMS error between input (acoustic) and output (electric) binaural cues were calculated per participant per condition. The interleaved strategy had the least error in delivering ITDs, while the clinical strategy had the most error in delivering ITDs. A linear mixed-effects model revealed a significant difference across conditions in RMS error between electric and acoustic ITDs [*χ*^2^(3) = 83.76, *p <* 0.001]. *Post hoc* tests revealed that all strategies were significantly different from each other except All-High and Best strategies. There was no difference across conditions in RMS error between electric and acoustic ILDs [*χ*^2^(3) = 5.0375, *p* = 0.17]. Similarly, there was no difference in localization error across conditions [*χ*^2^(3) = 3.1, *p* = 0.38], suggesting that ILDs and not ITDs underlie localization performance even with mixed rates strategies.

## 4 Discussion

In this study, we showed the first evidence of reliable delivery of real-time ITD cues in the free field, which was only made possible by CCi-MOBILE. Analysis of binaural cues demonstrates that mixed rates coding strategies preserved acoustic ITDs in electrical stimulation with some precision, as a result of synchronization and explicit encoding of ITD cues. These results represent significant advance in audio signal processing and embedded system design for auditory research. However, despite the improved encoding of ITDs, listeners were unable to achieve improved performance. We had hypothesized that good ITD sensitivity was a necessary requirement for CI listeners to benefit from mixed rates strategies in the free field. This hypothesis was based on the assumption that the sensitivity to ITDs was necessary to see any benefit from the strategy that provides ITD cues. For CI users with a CI in one ear and acoustic hearing in the contralateral ear, ITD thresholds were correlated with localization error (Gifford et al., 2014). However, we did not find evidence that supports the same hypothesis in our bilateral CI listeners. We found instead that the worst ITD thresholds were correlated with poor localization performance with the All-high strategy, suggesting that listeners with the lowest thresholds (i.e., most sensitive to ITDs) were already the best performers.Overall, the localization performance was similar in this study to previously reported reviews of sound localization error. Although in this study the error ranged from 11 degrees to 50 degrees, the median scores were close to 25 degrees, which is consistent with the literature (Anderson et al., 2022; Dorman et al., 2014; Dunn et al., 2008). Measurements of the output of each processing strategy revealed the potential benefit of encoding ITD cues into the stimulation patterns. This benefit did not appear to extend to ILDs. We indeed did not expect the synchronization to improve the ILD cues since ILD is not a timing cue. Much of this improvement in ITD encoding can be attributed to the use of synchronization and continuous interleaved sampling in CCi-MOBILE rather than N-of-M or spectral peak picking. N of M strategies can disrupt the encoding of ITD cues in processor output (Gray et al., 2021; Kan et al., 2018), so switching from clinical unsynchronized processors to CCi-MOBILE probably removed much of the jitter from N of M strategy. However, since the error between the input and output ITDs was significantly improved from the best strategy (1 low-rate channel) to the interleaved strategy (5 low-rate channels), the provision of low rate ITDs on five of 10 channels also contributed to improved accuracy in coding. We also observed improvement in ITD encoding with the All-high strategy, where ITDs were not explicitly encoded in the pulse timing. This improvement probably reflects the benefit of bilateral synchronization and the onsets of electrical pulse trains. Considering that ITDs in the envelope could be “recovered” from bandpass filtering (Heinz and Swaminathan, 2009), it is also possible that the improved envelope ITDs could aid with sound localization. Nevertheless, the localization performance did not indicate the utilization of envelope ITDs.

The accuracy of localization varied greatly between individuals and even between different speaker locations for the same individual (see Figure 6). The clinical strategy resulted in the widest performance range among all strategies: from the smallest RMS error (IDO, see Figure 5) to the largest RMS error (IAU) observed in this study under all four conditions. Participants, including IAU, IBF, IBL, ICM, ICP, IDL, IDM, benefited from mixed rates or synchronized stimulation in CCi-MOBILE compared to the unsynchronized clinical strategy. For IAJ, IBO, IBY, ICD, ICI, the clinical strategy resulted in performance similar to other strategies, except that the Best or the Interleaved mixed rates strategy was the worst condition, suggesting potential negativity from assigning one or too many channels for low-rate stimulation. However, for the participants, IDA and IDO, the clinical strategy was probably too good for the mixed rates strategies to surpass. The clinical strategy for these two participants demonstrated the smallest RMS errors in this study. For both participants, the Best or the Interleaved mixed rates strategy outperformed the All-high strategy, reaching a level similar to that with the clinical strategy. These results indicate that whether or not a patient can benefit from a mixed rates strategy in the free field also depends on how well they localize sounds with their own clinical processors. If a patient already does a good job localizing sounds with clinical processors, possibly relying mainly on ILD cues, the room for improvement from introducing access to ITD cues can be limited. This is probably due to the fact that ILDs remain the dominant cue in acute localization tests, even when low-rate ITDs are available (Klingel and Laback, 2022; van Hoesel et al., 2008).

Importantly, we show that electrical ILDs are much smaller cues than acoustic ILDs, consistent with Dorman et al. (2014). This is possibly due to the smaller dynamic range available to CI users with electrical stimulation and likely due to compression in the logarithmic mapping to the electrical range of stimulation. There was no significant difference in the error between the acoustic and electric ILDs across the different coding strategies we tested for localization, probably due to the large variability in ILDs measured from the electrodograms. Kelvasa and Dietz (2015) demonstrated with auditory modeling that ILDs were also the most likely binaural cue to predict localization performance. However, the variability in the ILDs provided by all conditions, even the clinical condition, suggest that ILD coding is still an unaddressed issue for CI users. This indicates that there is more room for improvement in localization if these very top performers, that is, IDA and IDO, start using ITD cues provided by the mixed rates strategies. In fact, in our recent direct stimulation, lateralization study, in which ILD cues were explicitly removed (Borjigin et al., 2024), these very top performers, i.e., IDA and IDO, showed improvement in lateralization using ITDs that were provided through mixed rates strategies. Combined, though the mixed rates strategy can reliably deliver ITD cues in electrical stimulation, these ITD cues may not translate into improved localization performance, likely due to limited exposure to ITD cues and the perceptual dominance of ILDs for sound localization in the free field. Providing BiCI listeners with adequate experiences with ITD cues may be necessary to shift their reliance from ILD dominance to a combined reliance on ILD and ITD cues in free field conditions. CCi-MOBILE, being much more compact than traditional research testing platforms, now enables participants to take home novel stimulation strategies such as a mixed rates strategy for extended experiences and long-term evaluation in real-world listening environments. In addition to the lack of training, the absence of localization benefit from mixed rates strategy can also be due to the spread of excitation. It is possible that the low-rate stimulation was “masked” by the current spread from the adjacent high-rate stimulation, although the stimulation sites were quite spaced out in our study (Middlebrooks, 2004).

There are additional considerations for interpreting this study. Due to the controlled nature of testing in a sound booth, while testing was in the free field, performance on this task may not generalize to everyday listening conditions. More complicated stimuli, with reflections and multiple sound sources, are likely to occur outside of the sound booth, making it difficult to generalize the results here without additional testing. Another potential limitation was the recruitment of only Cochlear CI recipients. This was a necessary constraint because at the time of testing, the CCi-MOBILE only supported Cochlear implants. However, the principles of the mixed rate strategy are feasible on other CI platforms with some adjustments for differences in how the internal devices generate pulses.

Future directions include conducting a new experiment in which ILDs are reduced in the free field (e.g., using low-pass filtered stimuli) to evaluate the benefit of ITDs for sound source localization. One thing to note is that the binaural cues were recorded by behind-the-ear (BTE) microphones of CCi- MOBILE. Although Cochlear and MED-EL CIs also use BTE microphones, Advanced Bionics provides in-the-canal microphones, which are called T mic. BTE microphones may offer less distinct and so less useful ILD cues than in-the-canal microphones such as T mics (Jones et al., 2016; Kolberg et al., 2020; Mayo and Goupell, 2020). Future steps also include measuring the acoustic inputs in the ear canal.

## Data Availability

The raw data supporting the conclusions of this article will be made available by the authors, without undue reservation.
